# Supplementary figures and images for: Role of SIRT3 in Microgravity Response: A New Player in Muscle Tissue Recovery
Source: Cells. 2023 Feb 22;12(5):691. doi: 10.3390/cells12050691 (PMC10000945; doi:10.3390/cells12050691)

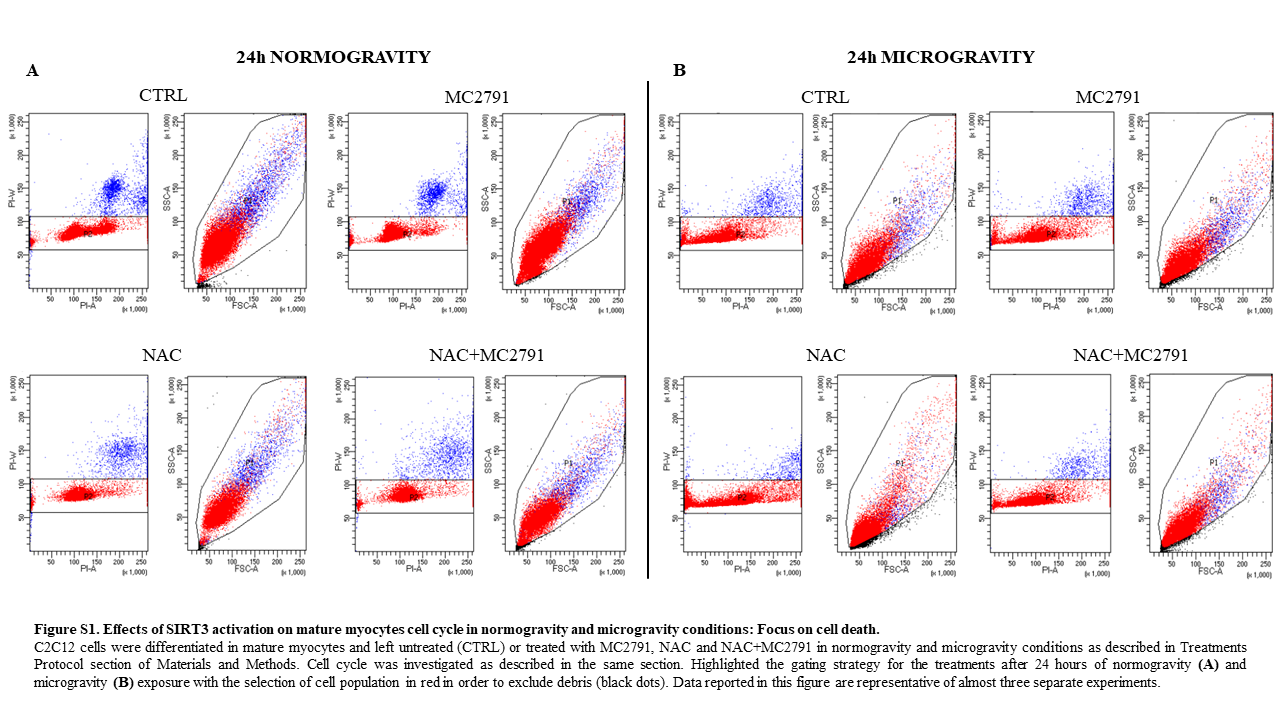

Supplement: Supplementary file 1 [file cells-12-00691-s001.zip › Figure S1.TIF]

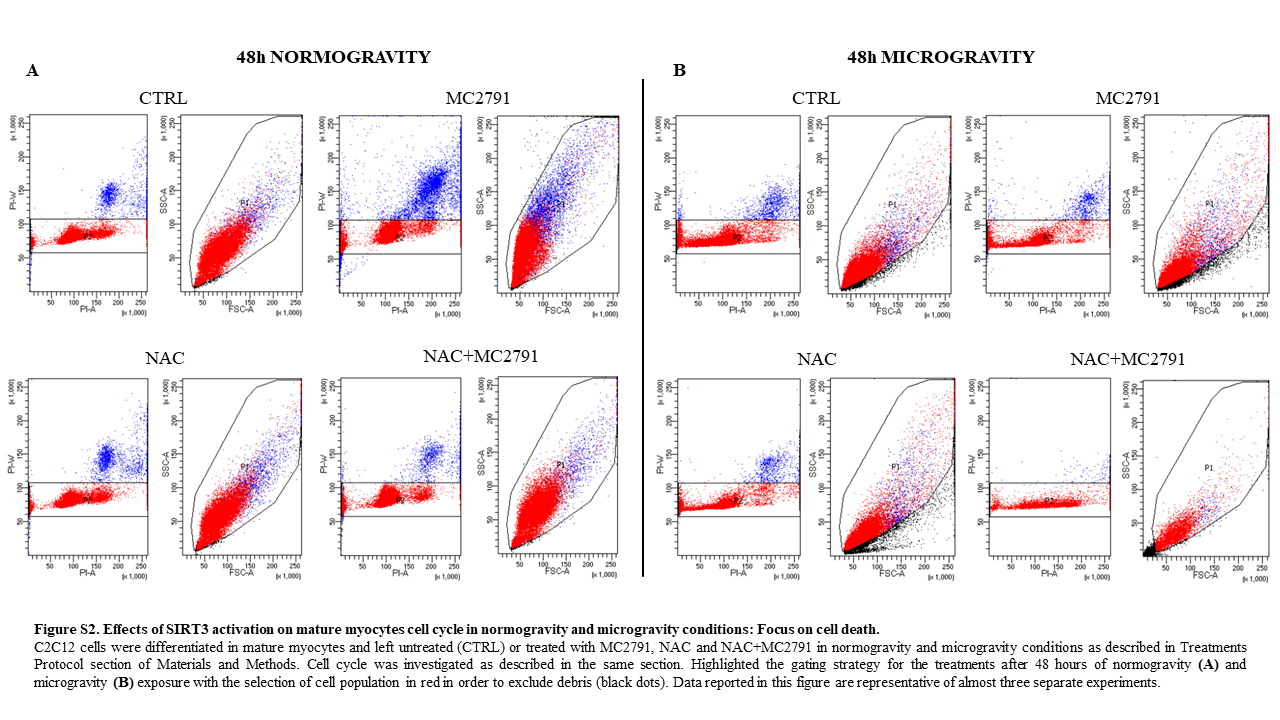

Supplement: Supplementary file 1 [file cells-12-00691-s001.zip › Figure S2.TIF]

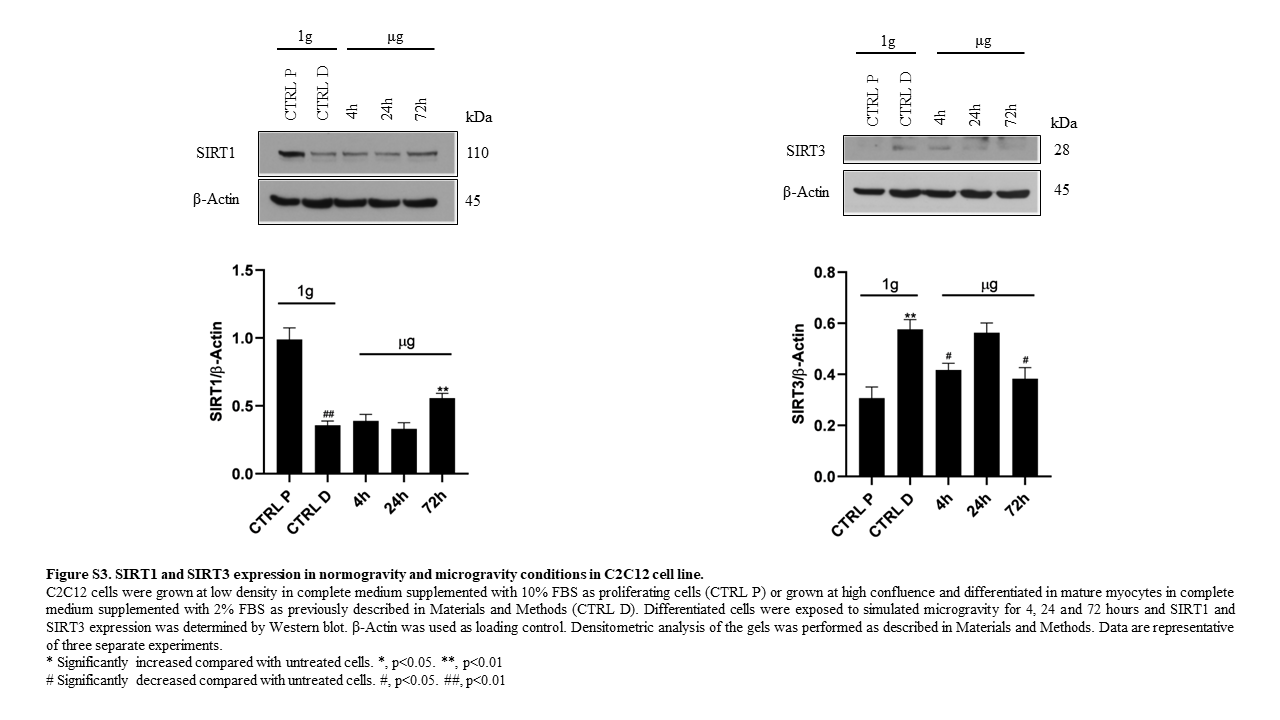

Supplement: Supplementary file 1 [file cells-12-00691-s001.zip › Figure S3.TIF]

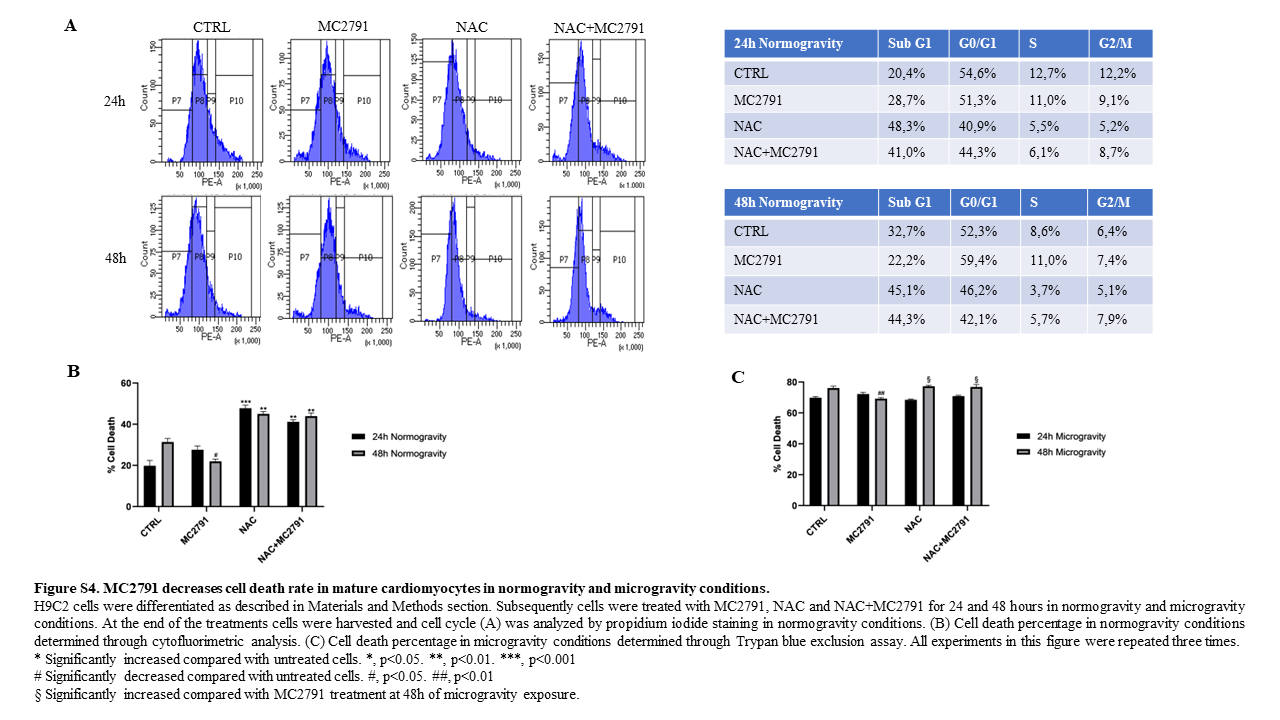

Supplement: Supplementary file 1 [file cells-12-00691-s001.zip › Figure S4.TIF]

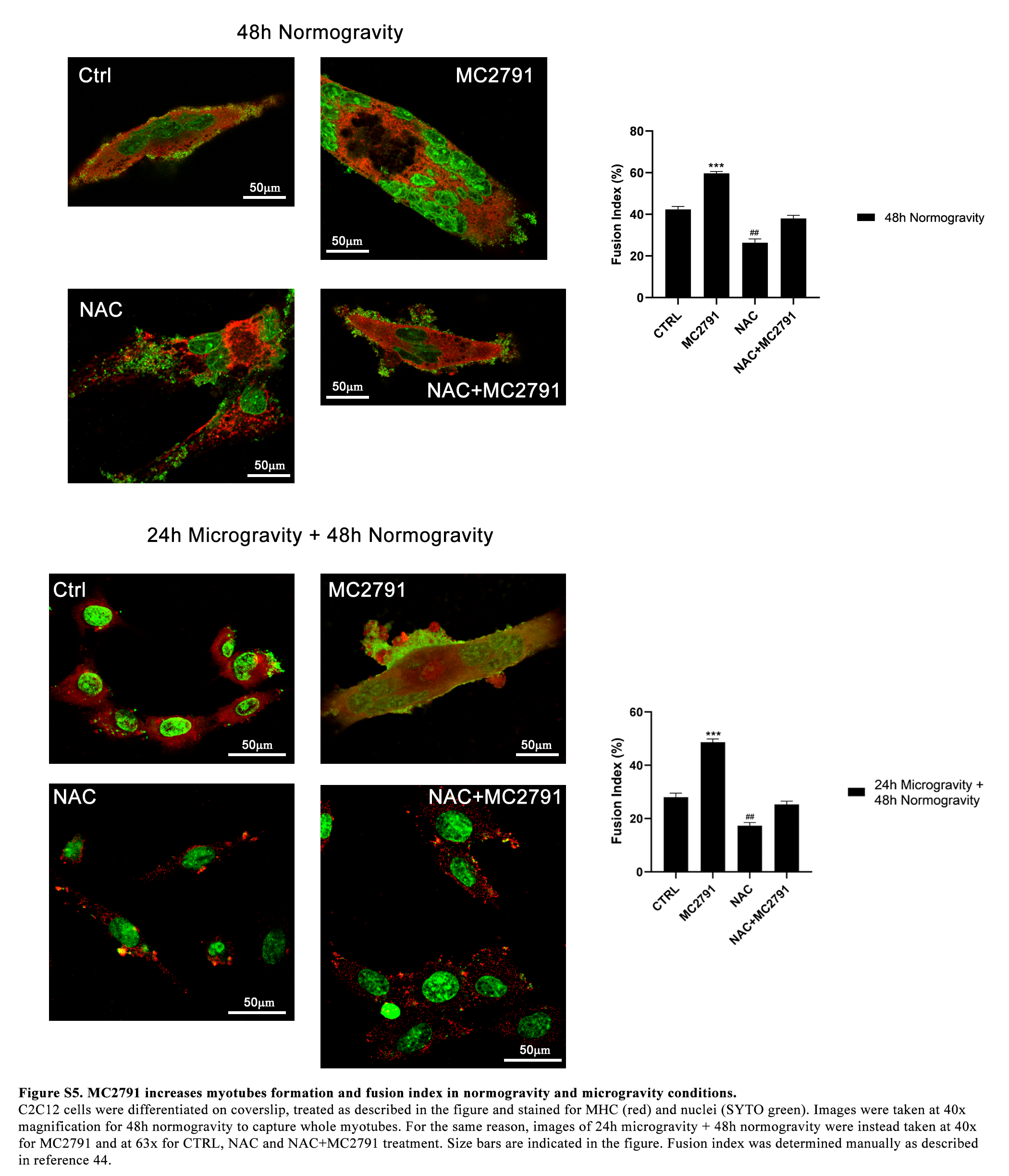

Supplement: Supplementary file 1 [file cells-12-00691-s001.zip › Figure S5.tif]

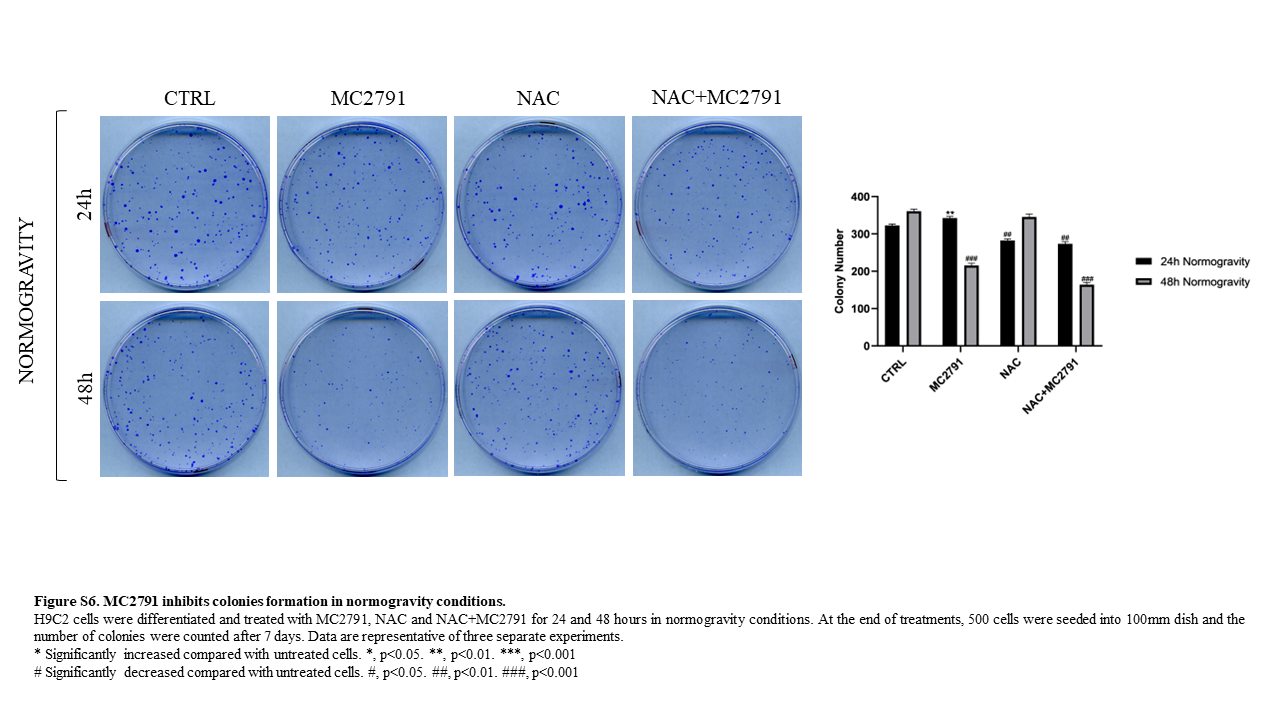

Supplement: Supplementary file 1 [file cells-12-00691-s001.zip › Figure S6.TIF]

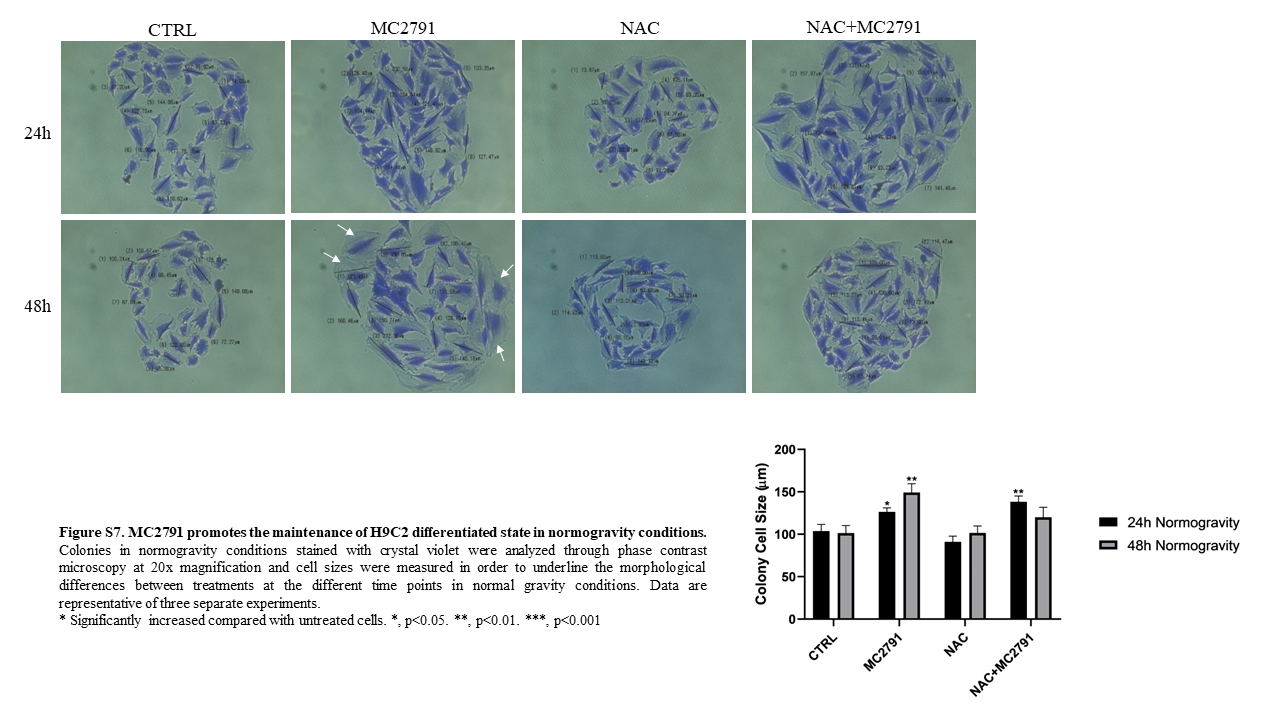

Supplement: Supplementary file 1 [file cells-12-00691-s001.zip › Figure S7.TIF]

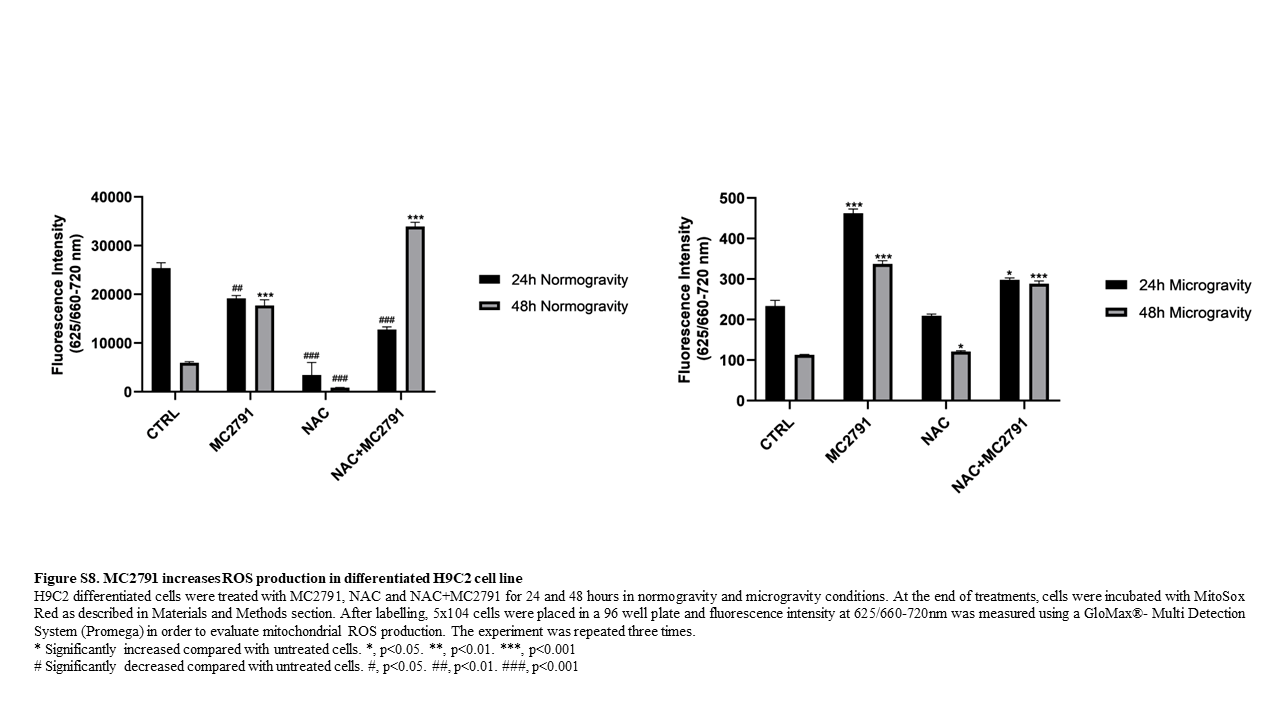

Supplement: Supplementary file 1 [file cells-12-00691-s001.zip › Figure S8.TIF]
